# Yield-Maturity Relationships of Summer Maize from 2003 to 2017 in the Huanghuaihai Plain of China

**DOI:** 10.1038/s41598-019-47561-2

**Published:** 2019-08-06

**Authors:** Jinzhong Yang, Yinchang Li, Hongbo Cao, Hongliang Yao, Wei Han, Shixian Sun

**Affiliations:** 1 Agronomy College/Shandong Key Laboratory of Rainfed Agriculture, Qingdao Agricultural University, Qingdao, 266109 P.R. China; 2 Agricultural Department of Shanxi Province, Taiyuan, 030001 P.R. China; 3 Agricultural Department of Shandong Province, Jinan, 250013 P.R. China; 4 The National Service Center for Agricultural Technology and Extension, Agriculture Ministry, Beijing, 100125 P.R. China

**Keywords:** Plant ecology, Plant physiology

## Abstract

Information on yield-maturity relationships is important for maize breeding and cultivation, but it is seldom available in geographic zones where there are limited heat resources for summer maize. Two novel systematic crop yield models were put forward in terms of production efficiency. These models as well as three other conventional models were used to analyze the crop yield and maturity dataset of 23,691 records that were collected from the annual reports for the national summer maize zonal trials conducted in the Huanghuaihai Plain of China during 2003 to 2017. (1) Crop yield increases were usually below 14.5 kg/666.7 m^2^ due to longer maturity days, varying from 1 d to 15 d increments. Maize hybrids with later maturity fell into five categories: statistically significantly less, not significantly less, the same, not significantly more, or statistically significantly more output than their earlier counterparts. (2) Three yield components acted on crop yield gaps in the order of descending effects as kernel number per ear ≈ 1000-kernel weight > ear number per unit land area. (3) Space production efficiency was more important than canopy volume to crop yield. (4) Time production efficiency was dominant and maturity was negligible in crop yield formation. The findings provide insights into yield–maturity relationships in maize and useful information for summer maize breeding and cultivation strategies.

## Introduction

Plant growth duration (maturity) is an important factor in the development of yield in maize (*Zea mays L*). Prolonged maturity makes more days available for canopy photosynthesis, leading to higher biomass production and thus higher crop yield. Maize production in China is affected by the global warming induced by the greenhouse effect. Several studies have reported on what has happened to maize maturity in China. Meng^1^ and Di^2^ found that the main phenological stages of summer maize are delayed significantly in Beijing-Tianjin-Hebei Region and Shandong Province, but they are advanced in Henan Province based on phenological records for summer maize from agro-meteorological stations during 1989–2001 in the Huanghuaihai Plain. Maize maturity increased at an average rate of 2.72 d 10 a^−1^ with the highest rate of 3.36 d 10 a^−1^ for Beijing-Tianjin-Hebei Region. Cui^3^ found that summer maize maturity was delayed in 64% of agro-meteorological stations during 2000–2013 and winter wheat was delayed in 78%. Contrary to expectations, however, average temperatures during the growth period of summer maize did not show an obvious increasing or decreasing trend. Days to maturity of maize increased from 120.9 d in 2002 to 133.9 d in 2009 in Northeastern China. At the same time, however, crop yields for Liaoning Province and Heilongjiang Province decreased by 1.72% and 14.5%, respectively, while Jinlin and Inner Mongolia yielded more by 18.9% and 2.8%, respectively, compared to year 2002^4^.

Meanwhile, there is limited information on yield-maturity relationships in maize in recent years, especially in the summer maize zones of China during climatic warming. Insights into yield changes induced by maturity differences, such as how individual crop yield components contribute to yield changes, may help in breeding programs and cultivation practices. Based on the dataset of 23,691 records from the national summer maize zonal trials conducted in the Huanghuaihai Plain of China during 2003 to 2017, the objectives of this paper are: (1) to propose and validate two novel production efficiency models for cropping systems; (2) to determine whether and how maturity days of hybrids affect maize crop yield; (3) to compare model components for the yield effect sizes associated with maturity days by means of several systematic models; and (4) to evaluate the relative importance of the gaps in model components to maize crop yield changes with respect to maturity days.

## Models, Data and Methods

### Six models from a systematic viewpoint decompose crop yield into different components

Usually, maize crop yield is divided into three components in one equation (Eq. 1), which was referenced as three-part model.

1 $${\rm{Crop}}\,{\rm{yield}}={\rm{ear}}\,{\rm{number}}\,{\rm{per}}\,{\rm{unit}}\,{\rm{land}}\,{\rm{area}}\,\ast \,{\rm{kernel}}\,{\rm{number}}\,{\rm{per}}\,{\rm{ear}}\,\ast \,{\rm{weight}}\,{\rm{per}}\,{\rm{kernel}}$$

We collapse (kernel number per ear * weight per kernel) in Eq. 1 in order to get a model at the ear level (Eq. 2).

2 $${\rm{Crop}}\,{\rm{yield}}={\rm{ear}}\,{\rm{number}}\,{\rm{per}}\,{\rm{unit}}\,{\rm{land}}\,{\rm{area}}\,\ast \,{\rm{kernel}}\,{\rm{weight}}\,{\rm{per}}\,{\rm{ear}}$$

We collapse (ear number per unit land area * kernel number per ear) in Eq. 1 in order to get a model at the kernel level (Eq. 3).

3 $${\rm{Crop}}\,{\rm{yield}}={\rm{kernel}}\,{\rm{number}}\,{\rm{per}}\,{\rm{unit}}\,{\rm{land}}\,{\rm{area}}\,\ast \,{\rm{weight}}\,{\rm{per}}\,{\rm{kernel}}$$

Treating space as a measurable resource for the cropping system, and letting space production efficiency equal crop yield divided by canopy volume, namely yield per unit volume. Thus we get Eq. 4, referenced as space efficiency model.

4 $${\rm{Crop}}\,{\rm{yield}}={\rm{canopy}}\,{\rm{volume}}\,\ast \,{\rm{yield}}\,{\rm{per}}\,{\rm{unit}}\,{\rm{volume}}$$

where canopy volume equals to canopy height (i.e., plant height) times unit land area.

Treating time as a measurable resource for the cropping system, and letting time production efficiency equal crop yield divided by maturity days, namely daily yield. Thus Eq. 5 is referenced as the time efficiency model.

5 $${\rm{Crop}}\,{\rm{yield}}={\rm{maturity}}\,{\rm{days}}\,\ast \,{\rm{daily}}\,{\rm{yield}}$$

Sharing equally an assembly of resources, such as water, nutrients, etc., among all maize plants in cropping systems, and letting share production efficiency equal crop yield divided by plant density, namely yield per plant. Thus we get Eq. 6 (Yang, 2013).

6 $${\rm{Crop}}\,{\rm{yield}}={\rm{plant}}\,{\rm{density}}\,\ast \,{\rm{yield}}\,{\rm{per}}\,{\rm{plant}}$$

### Data collection

Data to be analyzed were extracted from all the yearly reports for the national summer maize zonal trials^5^, which were conducted in the Huanghuaihai Plain of China from 2003 to 2017 except for year 2010, in which no records are available with details for site by cultivar combinations. There were altogether 23,691 records in the data set. Extracted variables were as follows: site, plant density, group, cultivar, crop yield, maturity, plant height, ear kernel weight and 1000-kernel weight. A few of the records in the reports were incomplete. If so, the record was retained and the missing values were assigned to affected variables.

Tested maize cultivars’ growth duration varied from 76 to 121 days (Table 1), with a median of 101 days.

**Table 1:** Maize maturity observations and their occurrence frequency, 2003–2017.

Maturity, d	Count	Maturity, d	Count	Maturity, d	Count	Maturity, d	Count
76	1	87	92	98	1495	109	691
77	11	88	109	99	1504	110	518
78	10	89	213	100	1464	111	445
79	16	90	248	101	1527	112	262
80	8	91	378	102	1591	113	103
81	6	92	444	103	1484	114	65
82	1	93	620	104	1587	115	52
83	14	94	811	105	1350	116	29
84	24	95	920	106	1048	117	17
85	34	96	1311	107	953	118	14
86	45	97	1323	108	809	121	1

### Analysis of variables quantifying changes introduced by different maturity days

In order to compare cultivars with different maturity days, they were divided into two groups, namely, *A* and *B*. Suppose that group *A* matured *k* days later than *B*. The change between the two groups in a variable, for example, crop yield was computed as: change _*k*_ = *W*_*A*_ − *W*_*B*_. This formula was applied to all variables that were analyzed, including those extracted from the national reports and the newly computed ones in the six models described in the previous section.

Within every site by cultivar combination, for a given *k*, any cultivar from group *A* was compared against all cultivars from group *B* which ripened *k* days later. To avoid the repeated use of the same cultivars, comparisons between cultivar groups *A* and *B* were classified into two categories by maturity days. Cultivars of group *A* with even maturity days were in one category, and cultivars of group *A* with odd days were in the other category. The two categories were separately subject to follow-up statistical analysis.

### Statistical methods

All analysis variables stated above were subjected to the Wilcoxon signed rank test, to determine if maturity days affected that variable.

In order to estimate the share of crop yield changes for individual components, we introduced the interlocking substitution analysis, which is a popular method in economic analysis^6^. Take a three-part model (Eq. 1) as an example.

7 $$\begin{array}{rcl}X-{\rm{share}} & = & ({X}_{A}\mbox{--}{X}_{B})\,\ast \,{Y}_{B}\,\ast \,{Z}_{B}\\ Y-{\rm{Share}} & = & {X}_{A}\,\ast \,({Y}_{A}\mbox{--}{Y}_{B})\,\ast \,{Z}_{B}\\ Z-{\rm{Share}} & = & {X}_{A}\,\ast \,{Y}_{A}\,\ast \,({Z}_{A}\mbox{--}{Z}_{B})\end{array}$$

where *X*, *Y* and *Z* denote the individual components of crop yield that equals to *X* * *Y* * *Z*, and *A* and *B* denote different maturity days. *X*-share is component *X*’s contribution to the crop yield difference between *A* and *B*, and so on.

One may show that

8 $${W}_{A}\mbox{--}{W}_{B}=X-{\rm{share}}+Y-{\rm{share}}+Z-{\rm{share}}$$

where *W* denotes crop yield, *W*_*A*_ – *W*_*B*_ is the yield gap between cultivars with maturity days of A and B, and all other variables are the same as in Eq. 7.

To assess the relative importance of individual model components to crop yield differences between maturity days *A* and *B*, we computed their effect sizes of squared omega from ANOVA analysis of the yield changes shared by individual components^7,8^.

9 $${\omega }^{2}=\frac{d{f}_{effect}\times (M{S}_{effect}-M{S}_{error})}{S{S}_{total}+M{S}_{error}}$$

A different statistical method, i.e., multiple linear regression, was used to examine the relative importance of individual components. Also taking a three-part model (Eq. 1) as an example, the regression equation reads as:

10 $${W}_{A}\mbox{--}{W}_{B}={b}_{0}+{b}_{1}({X}_{A}\mbox{--}{X}_{B})+{b}_{2}({Y}_{A}\mbox{--}{Y}_{B})+{b}_{3}({Z}_{A}\mbox{--}{Z}_{B})$$

where the *b*’s are constants, and all other variables are the same as in Eqs 7 and 8.

To correct for the influences of different dimensions or units of individual yield components, their standard regression coefficients were used instead of the original *b*’s. The larger the absolute values of standard regression coefficients are, the larger the effects on crop yield changes. Robust statistical methods were utilized to eliminate the impacts of possible outliers and leverage points^9^. In addition, data records with maturity day gaps larger than 15 d were excluded because of low representativeness.

Regression analysis was separately applied to data associated with individual maturity spans from 1 d to 15 d for each of the even and odd maturity groups of longer days. Share efficiency models (Eq. 6) were not applied to data analysis because of the same values of plant density within the sites in the national maize trials.

## Results

### Descriptive statistics of maturity spans

Descriptive statistics were similar for both even and odd groups of maturity days, so their means are reported in Table 2. Differences in maturity days between different hybrids were spread widely among years, with maximum observations from 9 to 24 d. Twenty-five percent quantiles of the maturity gaps were 1 to 2 days, and medians were 2 to 3 days, while 99% quantiles varied from 7 to 14.5 days over the years. Thus, maturity spans larger than 15 days were seldom encountered, so they were discarded in the following analysis in order to ensure representativeness of analysis results.

**Table 2:** Quantile estimates of maturity spans between tested cultivars each year.

Year	Quantile, %														
	0	1	5	10	25	50	75	90	95	99	100	s^†^	cv^‡^	Skew.	Kurt.
2003	1.0	1.0	1.0	1.0	2.0	3.0	5.0	8.0	10.0	14.5	23.5	2.4	79.5	1.6	3.2
2004	1.0	1.0	1.0	1.0	2.0	3.0	5.0	8.0	9.5	13.0	19.5	2.4	79.5	1.3	1.6
2005	1.0	1.0	1.0	1.0	1.0	2.5	4.5	7.0	8.0	11.0	17.0	1.8	71.6	1.4	2.0
2006	1.0	1.0	1.0	1.0	1.0	2.5	4.0	7.0	9.0	12.5	21.5	1.8	71.6	1.9	4.8
2007	1.0	1.0	1.0	1.0	1.0	2.0	3.5	5.5	7.5	10.0	21.5	1.2	59.7	2.2	8.4
2008	1.0	1.0	1.0	1.0	1.0	2.0	3.0	4.0	5.5	7.5	9.5	1.2	59.7	1.6	2.7
2009	1.0	1.0	1.0	1.0	1.0	2.0	3.0	4.0	5.0	7.5	14.5	1.2	59.6	2.0	6.4
2011	1.0	1.0	1.0	1.0	1.0	2.0	3.0	4.0	5.0	9.0	13.5	1.2	59.6	2.7	11.8
2012	1.0	1.0	1.0	1.0	1.0	2.0	2.5	4.0	5.5	8.0	12.5	1.2	59.6	2.4	7.9
2013	1.0	1.0	1.0	1.0	1.0	2.0	3.0	4.5	6.0	8.5	12.5	1.2	59.6	1.9	4.0
2014	1.0	1.0	1.0	1.0	1.0	2.0	3.0	4.5	5.5	11.5	17.5	1.2	59.6	2.9	12.6
2015	1.0	1.0	1.0	1.0	1.0	2.0	3.0	4.0	5.0	7.0	10.5	1.2	59.6	1.6	3.1
2016	1.0	1.0	1.0	1.0	1.0	2.0	3.0	4.0	5.0	9.0	19.5	1.2	59.6	2.7	12.5
2017	1.0	1.0	1.0	1.0	1.0	2.0	3.0	5.0	6.0	8.5	15.0	1.2	59.6	1.7	4.1

^†^and ^‡^: Robust statistics for standard deviation and variation coefficient.

### Descriptive statistics and tests of crop yield changes introduced by different maturity spans

**Figure 1:**
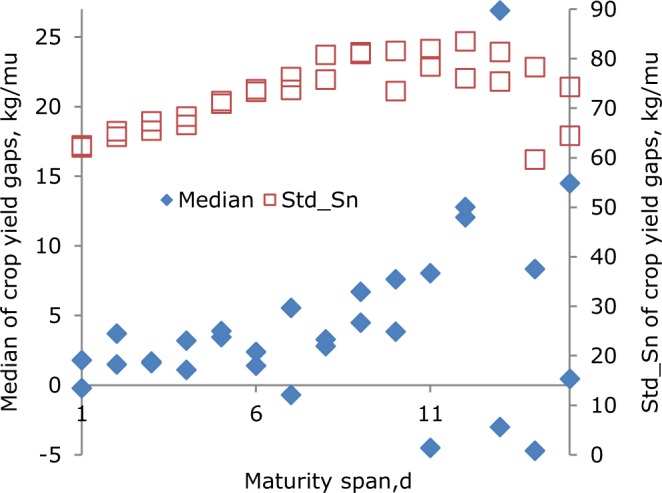
Robust statistics (median and standard deviation derived from *Sn*^30^) of crop yield gaps against maturity days span of even and odd days groups. *mu* denotes 666.7 m^2^ (the same below).

Median crop yield changes became larger with increased maturity spans, but with a few exceptions (Fig. 1). This means that a late maturity hybrid outpaced its early counterpart to a larger extent when their maturity span was enlarged. Crop yield changes were usually below 14.5 kg/666.7 m^2^ where the late hybrid matured 15 days later than its early counterpart, and most of them were less than 10 kg/666.7 m^2^. However, crop yield gaps varied greatly for the same span of maturity days. Standard deviations of crop yield gaps increased linearly with maturity span from 1 d to 8 d, and from 8 d on the spread of yield gaps remained stable at 81 kg/666.7 m^2^. Taking a maturity span of 12 d as an example, the variation coefficient of the yield gaps was 12.5/79.8 * 100% = 642.1%.

**Figure 2:**
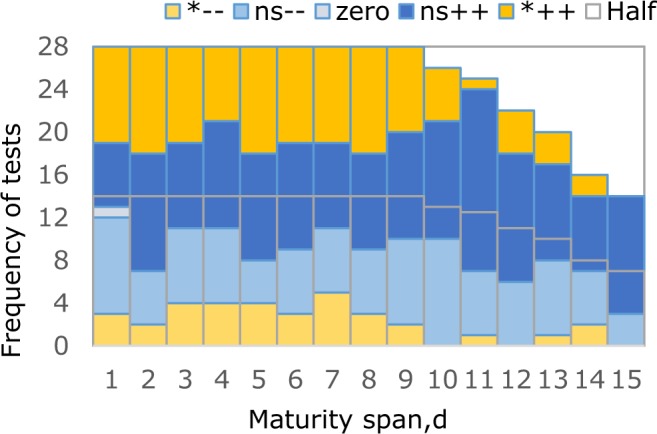
Frequency distribution of tests of crop yield gaps against maturity days span (Light grey lines mark half the number of tests for each maturity span. For each band, upward colour blocks denote yield gaps that are, in order, (1) statistically significant minuses, (2) not significant minuses, (3) zeros, (4) not significant pluses, (5) statistically significant pluses).

Figure 2 summarizes the sign rank tests of the effects of maturity days span on crop yield changes. Tests were divided into five categories for each of the 15 maturity spans: crop yield changes were either (i) negative and significant, (ii) negative but not significant, (iii) zero values, (iv) positive but not significant, or (v) positive and statistically significant. Average percentages of the five categories were 8.6%, 25.6%, 0.2%, 41.7% and 23.9%, respectively, out of 14 to 28 tests. The tests showed that the number of positive yield changes always exceeded of the number of negative ones. This demonstrated that late maturity hybrids generally produced higher yields than early hybrids with more chances. It is noteworthy that no matter how long the maturity span, almost one-third of comparisons between late and early maturity hybrids worked out to negative yield changes, i.e., late maturity hybrids output less yield than early ones.

### Interlocking substitution analysis of model components related to crop yield

#### Effect sizes on crop yield changes of the components of the three-part model

The squared omega values of effect sizes are useful indicators for relative effect importance. Table 3 shows that crop yield gaps were mainly attributed to both ear kernel numbers and kernel weights, and secondly to ear number per land area. Kernel number per ear generally affected yield changes to a greater extent than kernel weights. These patterns were repeated for most of the 15 different gaps of maturity days. In other words, no matter how many days maturity spans lasted, relationships remained almost the same between crop yield gaps and the gaps for its three components.

**Table 3:** Effect sizes (ω^2^) of three conventional components on crop yield changes during 2003–2017 national summer maize trials.

Maturity span, d	Ear number per area	Ear kernel number	1000-kernel weight
1	0.40	0.81	0.74
2	0.39	0.81	0.74
3	0.41	0.79	0.72
4	0.41	0.80	0.72
5	0.43	0.77	0.70
6	0.42	0.79	0.73
7	0.44	0.75	0.73
8	0.41	0.75	0.74
9	0.44	0.75	0.71
10	0.45	0.74	0.72
11	0.46	0.67	0.72
12	0.37	0.71	0.78
13	0.32	0.75	0.70
14	0.39	0.75	0.79
15	0.20	0.84	0.73

#### Effect sizes on crop yield changes of the components of the ear level model

The first contributor to crop yield gaps was ear kernel weight, the second was ear number per land area, with a mean effect size of 0.37 compared to 0.89 for ear kernel weight (Table 4).

**Table 4:** Effect sizes (ω^2^) of ear level components on crop yield changes during 2003–2017 national summer maize trials.

Maturity span, d	Ear number per area	Ear kernel weight	Maturity span, d	Ear number per area	Ear kernel weight
1	0.33	0.91	9	0.46	0.84
2	0.33	0.91	10	0.44	0.86
3	0.37	0.87	11	0.46	0.79
4	0.38	0.89	12	0.36	0.90
5	0.42	0.85	13	0.30	0.92
6	0.43	0.87	14	0.29	0.96
7	0.44	0.84	15	0.23	0.97

#### Effect sizes on crop yield changes of the components of the kernel level model

Table 5 shows that the first contributor to crop yield gaps was kernel number per land area, and the second was 1000-kernel weight, with a mean effect size of 0.74 compared to 0.87 for kernel number per land area.

**Table 5:** Effect sizes (ω^2^) of kernel level components on crop yield changes during 2003–2017 national summer maize trials.

Maturity span, d	Kernel number per area	1000-kernel weight	Maturity span, d	Kernel number per area	1000-kernel weight
1	0.92	0.75	9	0.90	0.72
2	0.92	0.74	10	0.89	0.74
3	0.93	0.73	11	0.89	0.72
4	0.93	0.72	12	0.82	0.83
5	0.93	0.69	13	0.78	0.74
6	0.91	0.70	14	0.80	0.86
7	0.90	0.74	15	0.83	0.66
8	0.88	0.75			

#### Effect sizes on crop yield changes of the components of the space efficiency model

Table 6 shows that the first contributor to crop yield gaps was yield per unit volume gap, and the second was canopy volume gap, with a mean effect size of 0.42 compared to 0.97 for yield per unit volume gap.

**Table 6:** Effect sizes (ω^2^) of space components on crop yield changes during 2003–2017 national summer maize trials.

Maturity span, d	Canopy volume	Yield per unit volume	Maturity span, d	Canopy volume	Yield per unit volume
1	0.55	0.98	9	0.36	1.00
2	0.53	0.98	10	0.37	1.00
3	0.50	0.99	11	0.36	0.96
4	0.47	0.99	12	0.34	0.97
5	0.44	0.99	13	0.29	0.93
6	0.42	0.99	14	0.51	0.89
7	0.44	0.98	15	0.34	0.85
8	0.39	0.99			

#### Effect sizes on crop yield changes of the components of the time efficiency model

Table 7 demonstrates that the dominant contributor to crop yield gaps was daily yield gap, and the effect size of maturity span was negligible, with a mean effect size of 0.01 compared to 0.93 for daily yield gap.

**Table 7:** Effect sizes (ω^2^) of time components on crop yield changes during 2003–2017 national summer maize trials.

Maturity span, d	Maturity	Daily yield	Maturity span, d	Maturity	Daily yield
1	0.00	0.95	9	0.01	0.92
2	0.00	0.95	10	0.02	0.89
3	0.00	0.95	11	0.02	0.93
4	0.00	0.96	12	0.02	0.90
5	0.01	0.96	13	0.03	0.92
6	0.01	0.95	14	0.02	0.83
7	0.01	0.96	15	0.02	0.91
8	0.01	0.95			

### Multiple linear regression analysis of crop yield divided into its components

#### Effects on crop yield changes of the gaps in components of the three-part model

Gaps of the three components in the three-part model were all positively correlative to crop yield gaps for each maturity span from 1 d to 15 d (Table 8). On average, a one gram increase in gaps of 1000-kernel weight raised crop yield gaps by 1.76 kg. A one kernel increase in gaps of ear kernel number increased crop yield gaps by 1.19 kg. A one ear addition in gaps of ear number per area land added crop yield gaps of 0.147 kg. Gaps of the three components acted on crop yield gaps in the order of descending effects as ear kernel number ≈1000-kernel weight > ear number per unit land area, as indicated by the standard regression coefficients.

**Table 8:** Regression coefficients and their standardized values of three conventional components for crop yield changes during 2003–2017 national summer maize trials^†^.

Regression	Item	Ear number per area gap	Ear kernel number gap	1000-kernel weight gap	R^2^
Coefficient	Min	0.114	1.01	1.62	0.67
Coefficient	Max	0.156	1.32	1.90	0.74
Coefficient	Mean	0.147	1.19	1.76	0.69
Standard coeff.	Mean	0.598	1.17	1.10	

^†^The analysis was separately applied to individual maturity spans from 1 d to 15 d for each of even and odd maturity groups of longer days. The same below.

#### Effects on crop yield changes of the gaps of components of the ear level model

Table 9 shows that both gaps of ear level components increased crop yield changes. Ear kernel weight had an average regression coefficient of 3.89 and revealed that every one additional gram of ear kernels led to an additional 3.89 kg increase in the crop yield gaps. Every one additional ear per land area raised crop yield gaps by 0.149 kg. The relative importance of the two components ranked as ear kernel weigh > ear number per land area according to their standard regression coefficients of 0.98 and 0.61.

**Table 9:** Regression coefficients and their standardized values of ear level components for crop yield changes during 2003–2017 national summer maize trials.

Regression	Item	Ear number per area gap	Ear kernel weight gap	R^2^
Coefficient	Min	0.134	3.52	0.70
Coefficient	Max	0.156	4.31	0.74
Coefficient	Mean	0.149	3.89	0.72
Standard coeff.	Mean	0.61	0.98	

#### Effects on crop yield changes of the gaps of components of the kernel level model

Table 10 shows that both gaps of kernel level components increased crop yield changes. On average, every 10,000 additional kernels per land area would increase the crop yield gap by 3.11 kg, and every one additional gram per 1000 kernels would increase the crop yield gap by 1.79 kg. Standard regression coefficients for the two different components were fairly close to each other. This means that both gaps of kernel number per land area and 1000-kernel weight had similar effects on crop yield changes.

**Table 10:** Regression coefficients and their standardized values of kernel level components for crop yield changes during 2003–2017 national summer maize trials.

Regression	Item	Kernel number per area gap	1000-kernel weight gap	R^2^
Coefficient	Min	2.52	1.66	0.68
Coefficient	Max	3.25	1.92	0.74
Coefficient	Mean	3.11	1.79	0.72
Standard coeff.	Mean	1.32	1.12	

#### Effects on crop yield changes of the gaps of components of the space efficiency model

Regression coefficients of the two space components were positive (Table 11). This means that an increase in either canopy volume gap or yield per unit volume gap would improve the crop yield gap. Every 10,000 m^3^ increase in gaps of canopy volume would increase crop yield gaps by 3283 kg, on average. A one kilogram increase in gaps of yield per cubic meters would increase the crop yield gaps by 1752 kg. Gaps of yield per unit volume contributed 51% more to crop yield gaps than the gaps of canopy volume when compared as standard regression coefficients: 1.31/0.87 = 1.51.

**Table 11:** Regression coefficients and their standardized values of space components for crop yield changes during 2003–2017 national summer maize trials.

Regression	Item	Canopy volume gap	Yield per unit volume gap	R^2^
Coefficient	Min	2803	1709	0.73
Coefficient	Max	3551	1804	0.78
Coefficient	Mean	3283	1752	0.75
Standard coeff.	Mean	0.87	1.31	

#### Effects on crop yield changes of the gaps of components of the time efficiency model

For each maturity span from 1 d to 15 d, the mean regression coefficient was 100, which indicates that a one kilogram increase in daily yield gap may, on average, increase the crop yield gap by 100 kg (Table 12). When pooled, the results show that the daily yield gap had a regression coefficient of 101 and that maturity span had a coefficient of 5.83, indicating the crop yield gap increased by 5.83 kg for every one day late for maturity. If compared by standard regression coefficients, the effects of maturity span is much greater than the daily yield gap.

**Table 12:** Regression coefficients and their standardized values of time components for crop yield changes during 2003–2017 national summer maize trials.

Regression	Item	Maturity span	Daily yield gap	R^2^
Coefficient	Min	—	96	0.74
Coefficient	Max	—	103	0.78
Coefficient	Mean	—	100	0.77
Coefficient	Pooled mean	5.83	101	0.99
Standard coef.	Pooled mean	0.106	1.02	

## Discussion

There have been numerous studies on yield-maturity relations in maize. The mid-season hybrid Norma and full-season hybrids of Maraton and Gazda were superior to the short-season hybrid Mara in maize dryland production^10^. The use of medium season hybrids is considered preferable to the short season ones because of higher yield and lower kernel moisture^11^. Yield usually increased with relative maturity. Yield increases with higher populations were greater for earlier hybrids than for later ones. To minimize yield reductions in dry years, relative maturities should not exceed 106 d for dryland corn in western Kansas of USA^12^. Environments dominated by late-season water stress caused yield reduction at high plant populations for one out of three early-maturing (95 to 99 d) hybrids and three late-maturing (114 to 118 d) hybrids. A well-adapted early-maturing hybrid can produce yields comparable to or better than late-maturing hybrids, particularly where late-season water stress is prevalent. However, the optimum plant population may be higher for early-maturing compared to late-maturing hybrids^13^. Short-season hybrids yielded less than the rest of the hybrids. Increments in the growth cycle length beyond that corresponding to intermediate hybrids, however, did not consistently increase grain yield^14^. In the area with limited heat resources, there was a tradeoff between a 1.4 bushel per acre increase in corn grain yield for every day increase in relative maturity and a 0.2 percent increase in grain moisture at the same time. In fact, there can be hybrids with shorter relative maturity that yield significantly better than longer day corn^15^. Early-season hybrids yielded more and matured earlier than mid-season ones under favorable conditions and high populations^16^. The prolonged maturity was not conducive to seed filling under the limited heat resources. The middle-early maturing hybrids yielded more^17^. Pearson’s correlations of fullsibs in families from three different maize varieties for yield and days to maturity found that two of the three were not significant, and the other one had only a weak positive correlative^18^.

Our results revealed that late maturity hybrids generally produced higher yields than early maturity hybrids with more chances, while late maturity hybrids yielded less than early ones in almost one-third of the cases. This means that many early maturity hybrids would yield equally or even more than late ones. What’s the reason for this? Stresses to the maize crop due to diseases, seasonal drought or heat shock might differentiate between early and late maturity hybrids, and many early maturity hybrids would escape from or tolerate stressful environments. On the other hand, there is the ever-increasing requirement for much lower moisture of kernels harvested by combination harvesters. So breeding summer maize for early maturity hybrids with a low moisture rate at harvest and high yield is more urgent than ever before in the Huanghuaihai Plain.

In summary, crop yield performances of different maturity groups in maize have displayed quite wide variations. We speculate that crop yield has a strong positive correlation with days to maturity of hybrids grown under optimum conditions, and a weak positive correlation under suboptimum conditions, and no correlation or a negative correlation under stressful conditions–especially in terms of heat and/or water resources. This guarantees further studies on yields under stressful conditions in the future.

Equation 4 resolves crop yield into the product of canopy volume by space production efficiency, i.e., yield per unit volume. Canopy space size, or canopy volume occupied by a crop population equals plant height times land area, so canopy volume is proportional to plant height. Many studies have found that plant height could be an indicator of non-grain and total biomass production for maize^19^. Few studies have related plant height to crop grain yield. Yang^20^ suggested a medium-plant hybrid was the ideal hybrid type for producing the highest crop yield based on multi-angle comparisons among maize hybrids of XY335 (high-plant), ZD958 (medium-plant) and GY1 (short-plant). Shi^21^ stated that hybrids with high-yielding capacity by means of high-populations are those hybrids whose plant height remains stable over wide plant population levels or remains stable after a limited increase with plant population, based on many years’ studies.

Our results showed that crop yield changes in relation to days to maturity was mainly due to yield per unit volume, or space production efficiency, and only secondarily due to space size (Table 6). This finding was supported by standard regression coefficients for these components (Table 11). So, maize crop yields are subject to space production efficiency rather than space size. Our findings imply that plant height is not a good indicator for crop yield in maize, even though it varied from 1.65 m to 3.55 m with a median of 2.64 m in the dataset used in this study (details not provided). The space efficiency model (Eq. 4) will facilitate simple and effective comparisons among maize genotypes with very different plant heights, such as inbred lines and their hybrids, in terms of space resources and their utilization. In a more broad sense, the model can be applied to compare different crop species such as maize, wheat, bean, and others.

Late maturity hybrids generally produced higher yields than early ones with more chances (Fig. 2). Generally, the canopy volume is larger for late maturity hybrids and the same is true for plant height because canopy volume is equal to plant height times land area (Figure S6 and Figure S9). Crop yield divided by canopy volume is equal to space production efficiency. Our data showed that late maturity hybrids had slightly higher space production efficiency (Figure S7).

Crop yield increases with plant density below the optimal plant density. Plant height may or may not increase with plant density, and it may even decrease with plant density^22^. Canopy volume is proportional to plant height. So, space production efficiency will be uncertain at the optimal plant density. The highest space production efficiency occurs at the optimal plant density only when plant height decreased or remained constant with plant density.

The growth duration for a crop includes time for crop development and yield formation. Time available for a crop is critical in agricultural areas with limited heat resources, such as the summer maize zone in the Huanghuaihai Plain. Time production efficiency, i.e., daily yield for one crop season, may lead to insights into the performance of cropping systems in terms of time consumption. Equation 5 provides an analytical tool of this kind. Our data revealed that the dominant contributor to maize yield changes was the daily yield gap, while the effect size of maturity span was negligible (Table 7). This finding was also supported by the fact that standard regression coefficients for maturity span and daily yield were 0.106 and 1.02 respectively (Table 12). This finding has implications for summer maize breeding programs. An implication of this is that much more attention should be paid to time production efficiency than to time length, i.e., days to maturity.

To our knowledge, this is the first study on cropping systems based on a time production efficiency model. Lindsey^23^ did not use the concept of time production efficiency, nor its theoretical model, even though they reported maize crop yield for short- and full-season hybrids. He found that short-season hybrids had a greater optimum plant population and lower maximum yield as compared to full-season hybrids. From their reported data, Table 13 was created to show that regardless of plant date or sites, short-season hybrids (CRM103) displayed higher time production efficiency than full-season ones (CRM112), increasing from 1.2% to 7.3%. However, crop yields for these maturity groups were in the reversed order. Nevertheless, one may infer that advantages in time production efficiency of short-season hybrids would become yield advantages in the presence of late-season drought, where the premature death is often the case in rainfed maize cropping systems. This deduction is verified by the fact that a well-adapted early-maturing hybrid such as Pioneer 3737 can produce yields comparable to or better than late-maturing hybrids, particularly where late-season water stress is prevalent^13^. However, the optimum plant population may be higher for early-maturing compared to late-maturing hybrids.

**Table 13:** Maize time production efficiencies for combinations among sites by planting dates by maturity groups (derived from Lindsey’s data, 2016).

Site	Month	Maturity group	Maximum yield, Mg hm^−2^	Time production efficiency	Difference %
NWARS	May	Short	11.48	7.43	7.3%
		Full	11.57	6.89	
	June	Short	11.12	7.20	2.8%
		Full	11.75	7.00	
WARS	May	Short	14.41	9.33	1.2%
		Full	15.48	9.22	
	June	Short	11.53	7.47	2.9%
		Full	12.17	7.25	
WST	May	Short	13.52	8.76	4.0%
		Full	14.11	8.40	
	June	Short	11.20	7.25	4.2%
		Full	11.67	6.95	

The crop yield model of three components (Eq. 1) provides a systematic approach for exploring causes of yield changes between late and early maturity groups of hybrids in maize. Results derived from two different analysis methods led to coincident conclusions that both kernel number per ear and 1000-kernel weight were the primary factors causing crop yield to change, and that ear number per land area was secondary, based on both arguments of squared omega values (Table 3) and standardized regression coefficients (Table 8). Results from both the ear level model (Tables 4 and 9) and the kernel level model (Tables 5 and 10) all showed consistent results, supporting our findings stated above. Plant densities in National Maize Trials varied from 3700 to 5000 p/666.7 m^2^ during 2003 to 2017, and plant densities within sites in one year were the same, except for year 2017 which included plant densities of 4500 and 5000. Effects of ear number per land area might be underestimated at the same plant density to some extent. However, when analyzing data for year 2017, the roles of the three yield components on yield gaps showed similar results (Table S1) to the data in Tables 3 and 8. A previous study provided findings consistent with the data in Tables 4 and 9. Specifically, the role of plant density was globally less than that of yield per plant (for maize, it can be considered as kernel weight per ear) under the condition that optimal plant densities varied from 2.95 to 9.62 plants per squared meters^24^.

Because the accumulated temperature is relatively insufficient for late maturity hybrids in the north region of Huanghuaihai Plain, the late maturity hybrids may not mature normally, which may affect the grain weight. The affected grain weight would strengthen the contribution of 1000-kernel weight to crop yield. However, the grain weight reported in this paper was not affected by the condition that the late maturity hybrids may not mature normally because all cultivars tested in National Trials are harvested after the physiologically mature stage.

According to the findings above, farmers growing summer maize are recommended to focus not only on cultivation practices to improve ear kernel number, but also practices to increase kernel weight. For summer maize breeders, traits relevant to stress resistance, especially in mid- and late- growth stages, are key targets for gene pyramiding and phenotype screening.

Liu found that the higher grain yield for the optimum leaf removal under high plant density was mainly due to higher kernel weight and more harvested ears in summer maize^25^. Wang and Li observed a slightly different pattern of yield components that both kernel number per ear and harvested ear number had almost the same direct effects on maize yield, and kernel weight contributed to crop yield to a smaller extent, based on 57 high-yielding (≥1.5 t/hm^2^) fields in China^26^. Milander reported that ear number per land area, kernel number per ear and kernel weight affected crop yield similarly for early-maturity hybrids while ear kernel number had the largest direct effect for the mid- and late-maturity hybrids^27^. DaNa indicated that the yield decreases due to temperature elevation were due more to the decrease in ear kernel number than to the decrease in kernel weight in two tested maize cultivars^28^. Zhang showed that a novel plant growth regulator significantly increased crop yield by 10.7% due to the increases of kernel weight by 3.2% and kernel number per ear by 4.4%^29^.

## Conclusion

Maximum differences in maturity between tested maize hybrids ranged from 9 to 24 d within years of the trials conducted during 2003–2017 in the Huanghuaihai Plain, along with medians of 2 to 3 d. Average yields of late maturity hybrids were higher than those of early ones, but the amounts of yield changes varied dramatically. Almost one-third of comparisons between late and early maturity hybrids showed negative yield changes, i.e., late maturity hybrids producing less yield than early ones.

Regardless of the differences in days to maturity, differences in maize crop yield induced by maturity changes were mainly due to both ear kernel numbers and kernel weights, and secondly to ear number per land area. At the ear level, ear kernel weight played a more prominent role in yields when compared to ear number per land area. At the kernel level, kernel number per land area exerted more impact on yields than kernel weight.

The space efficiency model provides an approach for understanding crop yield variations in cropping systems in terms of canopy space size or canopy volume and space production efficiency, i.e., yield per unit volume. Space production efficiency was always more important for maize crop yield than canopy space size, regardless of differences in days to maturity.

The time efficiency model treats time as a crop production resource and decomposes crop yield into the two components of time length and time production efficiency, i.e., daily yield. Maize yield changes contributed absolutely to daily yield changes while maturity changes were negligible for maize yield.

In summary, the findings featured multiple perspectives of yield and maturity relationships in the summer maize zones of Huanghuaihai Plain of China, and put forward two novel systematic crop yield models to facilitate new insights into cropping systems.

## Supplementary information

Supplementary information
