# Supplementary material for: Yield-Maturity Relationships of Summer Maize from 2003 to 2017 in the Huanghuaihai Plain of China
Source: Sci Rep. 2019 Aug 6;9:11417. doi: 10.1038/s41598-019-47561-2 (PMC6684628; doi:10.1038/s41598-019-47561-2)
Supplement: Supplementary file 1 — Supplementary information [file 41598_2019_47561_MOESM1_ESM.pdf]

Supplementary information

Yield-Maturity Relationships of Summer Maize from 2003 to 2017 in the Huanghuaihai Plain of China

Jinzhong YANG, Yinchang LI, Hongbo CAO, Hongliang YAO, Wei HAN, Shixian SUN

Contents:

Table S1. Effect sizes (ω^2) of three conventional components on crop yield changes in 2017 national summer maize trial

Figure S1. Accumulated frequency distribution of tests for gaps in ear number per area against maturity days span

Figure S2. Accumulated frequency distribution of tests for gaps in kernel number per ear against maturity days span

Figure S3. Accumulated frequency distribution of tests for gaps in 1000-kernel weight against maturity days span

Figure S4. Accumulated frequency distribution of tests for gaps in kernel weight per ear against maturity days span

Figure S5. Accumulated frequency distribution of tests for gaps in kernel number per area against maturity days span

Figure S6. Accumulated frequency distribution of tests for gaps in canopy volume against maturity days span

Figure S7. Accumulated frequency distribution of tests for gaps in yield per unit volume against maturity days span

Figure S8. Accumulated frequency distribution of tests for gaps in daily yield against maturity days span

Table S1. Effect sizes (ω^2) of three conventional components on crop yield changes in the 2017 national summer maize trial

Maturity span, d	Ear number per area	Ear kernel number	1000-kernel weight
1	0.37	0.86	0.76
2	0.33	0.85	0.75
3	0.42	0.79	0.75
4	0.37	0.84	0.71
5	0.46	0.78	0.70
6	0.50	0.81	0.70
7	0.45	0.73	0.57
8	0.48	0.70	0.62
9	0.54	0.67	0.33
10	0.43	0.74	0.60
11	0.56	0.38	0.30
12	-	-	-
13	-	-	-
14	-	-	-
15	-	-	-

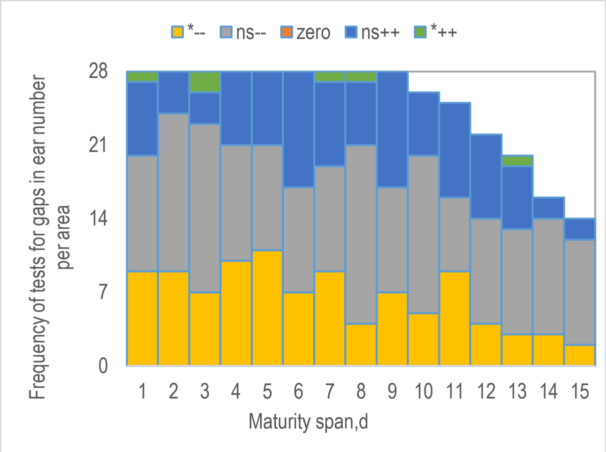

Figure S1. Accumulated frequency distribution of tests for gaps in ear number per area against maturity days span (For each band, upward colour blocks denote the gaps that are, in order, statistically significant minuses, or not significant minuses, or zeros, or not significant pluses, or statistically significant pluses)

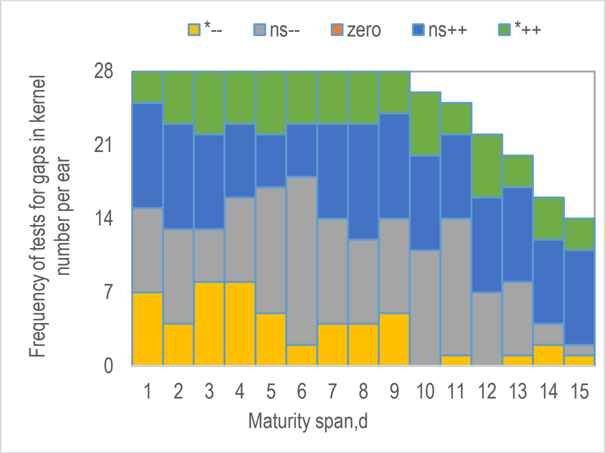

Figure S2. Accumulated frequency distribution of tests for gaps in kernel number per ear against maturity days span (For each band, upward colour blocks denote the gaps that are, in order, statistically significant minuses, or not significant minuses, or zeros, or not significant pluses, or statistically significant pluses)

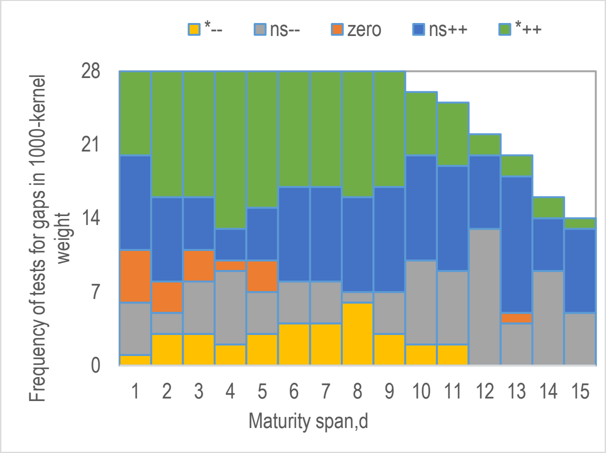

Figure S3. Accumulated frequency distribution of tests for gaps in 1000-kernel weight against maturity days span (For each band, upward colour blocks denote the gaps that are, in order, statistically significant minuses, or not significant minuses, or zeros, or not significant pluses, or statistically significant pluses)

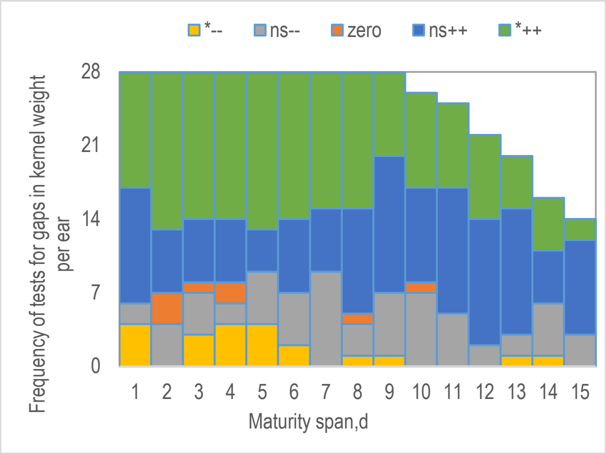

Figure S4. Accumulated frequency distribution of tests for gaps in kernel weight per ear against

maturity days span (For each band, upward colour blocks denote the gaps that are, in order, statistically significant minuses, or not significant minuses, or zeros, or not significant pluses, or statistically significant pluses)

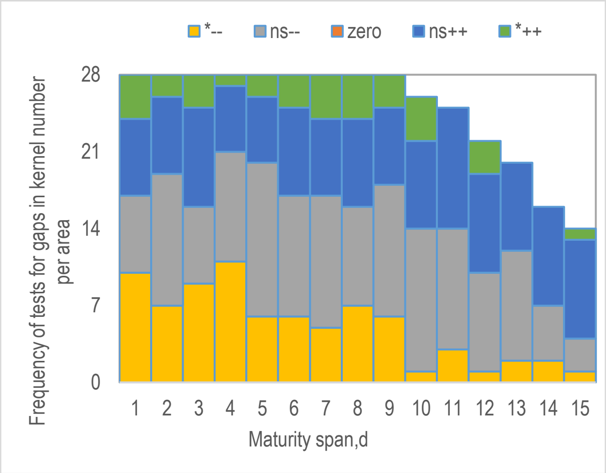

Figure S5. Accumulated frequency distribution of tests for gaps in kernel number per area against maturity days span (For each band, upward colour blocks denote the gaps that are, in order, statistically significant minuses, or not significant minuses, or zeros, or not significant pluses, or statistically significant pluses)

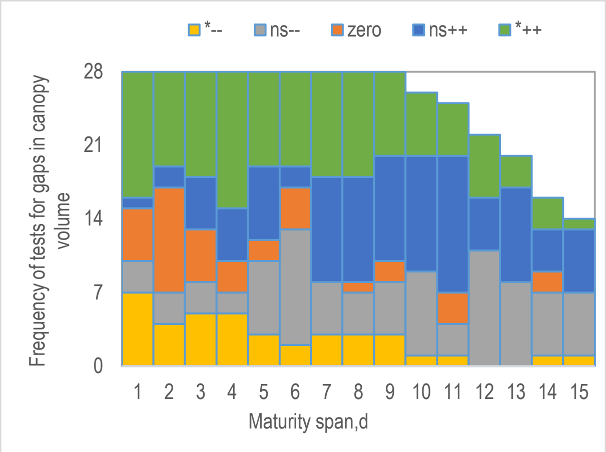

Figure S6. Accumulated frequency distribution of tests for gaps in canopy volume against maturity days span (For each band, upward colour blocks denote the gaps that are, in order, statistically significant minuses, or not significant minuses, or zeros, or not significant pluses, or statistically significant pluses)

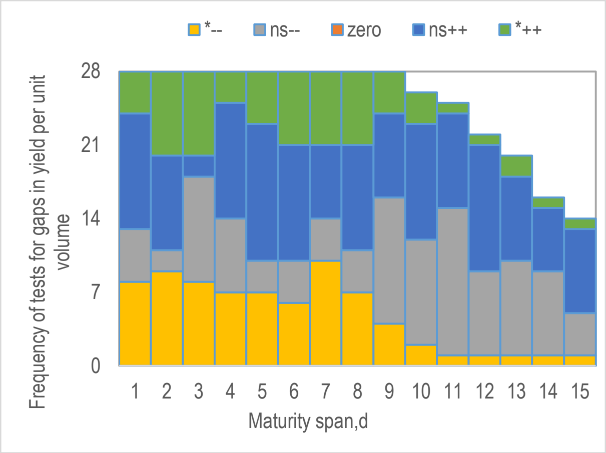

Figure S7. Accumulated frequency distribution of tests for gaps in space production efficiency, i.e., yield per unit volume, against maturity days span (For each band, upward colour blocks denote the gaps that are, in order, statistically significant minuses, or not significant minuses, or zeros, or not significant pluses, or statistically significant pluses)

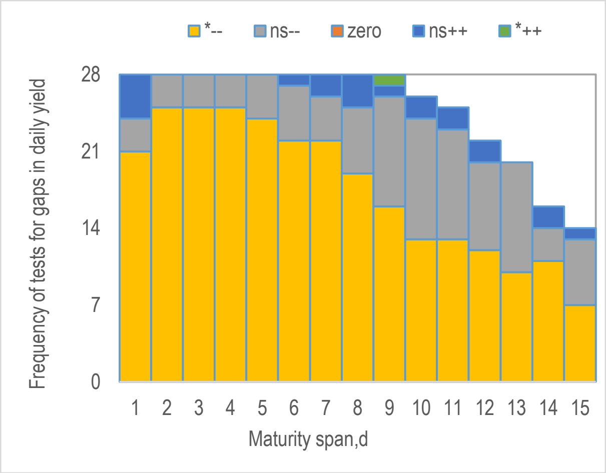

Figure S8. Accumulated frequency distribution of tests for gaps in time production efficiency, i.e., daily yield, against maturity days span (For each band, upward colour blocks denote the gaps that are, in order, statistically significant minuses, or not significant minuses, or zeros, or not significant pluses, or statistically significant pluses)

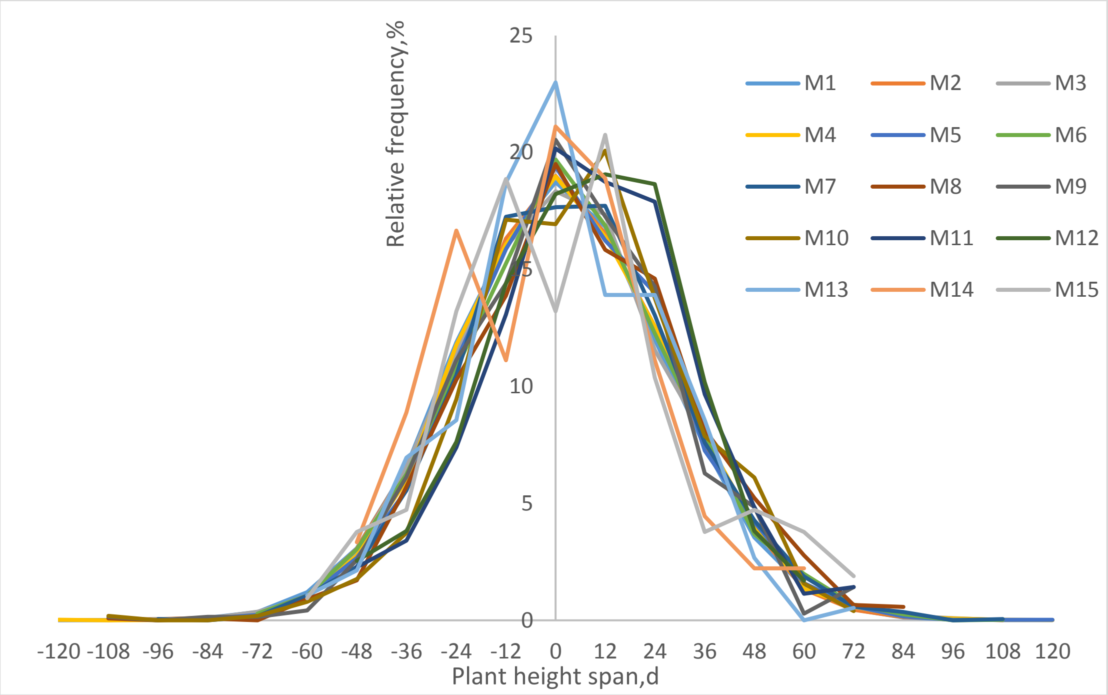

Figure S9. Histograms of differences in plant height in summer maize for individual spans of growth duration length (M15 denotes that late maturity cultivars ripen 15 days later than early ones. M14,…,and M1 follow the same logic)
